# Relationship Between Health Insurance Status and Frequency of Routine Medical Checkups

**DOI:** 10.7759/cureus.87847

**Published:** 2025-07-13

**Authors:** Feyisayo O Oguntuase, Consolata Uzzi, Tochukwu W Okahia, Opemipo Adetifa, Chinonso F Eziechi, Okelue E Okobi, Omamuyovbi F Nwoagbe, Oluwatayo A Dare

**Affiliations:** 1 General Medicine, National Pirogov Memorial Medical University, Vinnytsia, UKR; 2 Family Medicine, American University of Antigua, New York, USA; 3 Psychiatry, Leeds and York Partnership NHS Foundation Trust, Leeds, GBR; 4 Family Medicine, Kyiv Medical University, Calgary, CAN; 5 Public Health, Liberty University, Lynchburg, USA; 6 Family Medicine, Larkin Community Hospital Palm Springs Campus, Hialeah, USA; 7 Family Medicine, IMG Research Academy and Consulting LLC, Homestead, USA; 8 Internal Medicine, University of Port Harcourt Teaching Hospital, Port Harcourt, NGA; 9 Psychiatry, Foothills Medical Clinic, Calgary, CAN

**Keywords:** brfss, health disparities, health insurance, income, preventive care, race, routine check-up, sex, us adults

## Abstract

Background: Routine medical checkups are essential for early disease detection and prevention. However, disparities in utilization persist across sociodemographic groups, particularly in relation to health insurance coverage in the US population.

Objective: This study aims to examine the relationship between health insurance status and recent routine medical checkups among US adults, using nationally representative survey data.

Methods: This cross-sectional study analyzed data from the 2019 Behavioral Risk Factor Surveillance System (BRFSS) (n = 329,549; weighted population = 198,183,089). Descriptive statistics, chi-square tests, and survey-weighted logistic regression were employed to examine the associations between recent checkup status and various variables, including insurance coverage, age, sex, education, income, and race/ethnicity.

Results: Individuals with health insurance had nearly four times the odds of having had a recent checkup compared to those without insurance (OR = 3.90, 95% CI: 3.69-4.12). Female sex, older age, and Hispanic or Black race/ethnicity were also positively associated with recent checkups. Conversely, lower income and educational attainment were linked to reduced utilization.

Conclusion: Health insurance coverage is a strong predictor of routine healthcare utilization. Expanding access to insurance may substantially improve the uptake of preventive services, particularly among underserved populations.

## Introduction

Access to healthcare is a significant determinant of population health and well-being in the United States. Despite technological advancements and healthcare reforms aimed at improving health services, disparities in preventive care utilization persist [1]. Among various factors influencing healthcare use, health insurance coverage remains one of the most significant predictors [2]. A commonly used indicator of healthcare access is the frequency of routine medical checkups [3], with annual visits recommended for early detection, timely treatment of chronic conditions, and overall health maintenance [4,5].

In the United States, access to health coverage is often linked to employment or specific insurance programs, making insurance beneficial both financially and psychologically. Individuals with insurance are more likely to have a regular source of care, receive medical guidance, and engage in preventive services such as screenings, immunizations, and annual checkups [6,7]. Conversely, those without insurance may forgo medical care due to financial constraints or limited awareness, resulting in delayed diagnoses and poorer health outcomes, such as later-stage cancer detection [8,9].

Health insurance plays a pivotal role in addressing disparities in preventive care utilization. The implementation of the Affordable Care Act (ACA) in 2010expanded Medicaid eligibility in many states and established health insurance marketplaces [10,11], significantly improving coverage among historically underserved populations [12]. Nonetheless, gaps remain: many Americans remain uninsured, while others possess limited coverage that fails to meet their healthcare needs [13].

Routine medical checkups are a main part of preventive care, enabling early intervention for conditions like hypertension, diabetes, and various cancers. These visits typically include physical examinations, screening tests, and personalized counseling to assess and mitigate health risks [14]. The Centers for Disease Control and Prevention (CDC) and the United States Preventive Services Task Force (USPSTF) emphasize regular checkups as a strategy for reducing the burden of chronic diseases [15]. Consistent preventive care is associated with reduced healthcare costs, lower hospitalization rates, and improved long-term health outcomes [16].

Despite the advantages of regular medical checkups, low-income individuals remain less likely to seek routine medical care. Barriers such as lack of insurance, financial hardship, limited health literacy, and geographic inaccessibility hinder utilization of preventive services [17]. Additionally, provider shortages in certain regions and cultural or language barriers may limit access to care [18]. Understanding how insurance coverage interacts with these barriers is essential for designing policies that promote health equity and expand healthcare access [19]. This study uses data from the 2019 Behavioral Risk Factor Surveillance System (BRFSS), collected between January and December 2019, to examine the association between health insurance status and utilization of routine medical checkups among US adults [20,21]. The primary objective is to assess whether having health insurance is associated with a higher likelihood of receiving a routine checkup within the past 12 months.

## Materials and methods

Study design and data source

This cross-sectional study analyzed data from the 2019 BRFSS, a nationally representative, annual telephone survey administered by the CDC [21]. The BRFSS collects data on health-related risk behaviors, chronic health conditions, and use of preventive services among non-institutionalized US adults aged 18 years and older. For the 2023 cycle, both landline and cellular telephone interviews were conducted across all 50 states, the District of Columbia, and participating US territories, ensuring robust geographic representation.

Study population

The analytic sample included adult respondents (aged ≥ 18 years) with complete data on key variables: routine medical checkup (CHECKUP1), health insurance coverage status (HLTHPLN1), and relevant sociodemographic characteristics, including age (continuous), sex (male/female), race/ethnicity (NH White, NH Black, Hispanic, etc.), education (highest level completed), and income (categorized annual household income). Respondents who had missing or refused responses for these variables were excluded using list-wise deletion to preserve data integrity.

Variables and operational definitions

The primary outcome variable in this study was the timing of the most recent routine medical checkup, as measured by the BRFSS variable CHECKUP1. Responses were dichotomized to indicate whether the respondent had a routine checkup within the past 12 months (categorized as “recent checkup”) or had not (including those who reported a checkup more than a year ago or never). The main independent variable was health insurance status, derived from the HLTHPLN1 variable, which assessed whether respondents currently had any form of healthcare coverage. Those who responded “Yes” were classified as insured, while those who responded “No” were considered uninsured. Several covariates were included to control for potential confounding. Age was treated as a continuous variable using the AGE field. Sex was categorized based on the binary classification provided in the SEX variable (male or female). Race and ethnicity were determined using the RACEGR3 and HISPANC2 variables, which were combined to create mutually exclusive racial/ethnic categories. Socioeconomic status was represented by two variables: income (measured by INCOME2, which categorized annual household income) and education (assessed using the EDUCA variable, which captured the highest level of formal education completed).

Statistical analysis

Prior to analysis, the dataset was reviewed for completeness. Cases with missing values on any of the key analytic variables, including health insurance status, routine checkup status, race/ethnicity, education, income, and survey design variables, were excluded from the final analytic sample. This complete case analysis approach was justified due to the minimal proportion of missing data in most variables (<0.01%), except for household income, which had approximately 18% missingness. Given the large overall sample size of the BRFSS 2019 (n = 401,981), the exclusion of incomplete cases preserved sufficient statistical power while enhancing the interpretability and consistency of the multivariable models. No imputation procedures were employed.

All analyses were conducted using Stata 18 (StataCorp LLC, College Station, TX, US). To account for the complex sampling design of the BRFSS, including weighting, stratification, and clustering, survey weights (_LLCPWT) were applied using Stata’s svy commands.

Descriptive statistics summarized the distribution of variables. Bivariate associations between health insurance status and routine checkup behavior were assessed using weighted chi-square tests. A multivariable logistic regression model was then fitted to estimate the adjusted odds ratios (AORs) and 95% confidence intervals (CIs) for the likelihood of having had a routine medical checkup in the past year, comparing insured and uninsured respondents, while controlling for all listed covariates.

Model diagnostics, including tests for multicollinearity and goodness-of-fit, were conducted to assess model validity. A two-sided p-value < 0.05 was considered statistically significant.

Ethical considerations

The BRFSS data are publicly available, de-identified datasets collected by the CDC, and as such, the current analysis did not require institutional review board (IRB) approval. All data were used in accordance with the CDC’s data use guidelines. No identifiable personal health information was accessed or analyzed.

## Results

Table 1 shows the weighted sociodemographic characteristics stratified by health insurance status.

**Table 1:** Weighted sociodemographic characteristics by insurance status (n = 329,549; population size = 198,183,089) Values are survey-weighted using the BRFSS 2019 complex sampling design. Categorical variables were compared using design-based F-tests, and continuous variables (e.g., age) using survey-weighted t-tests. All p-values are two-sided. Asterisks indicate statistical significance: p < 0.05 (*), p < 0.01 (**), p < 0.001 (***). Comparisons reflect differences between insured and uninsured individuals. HS: high school.

Characteristic	Total (weighted %)	Insured (N = 173,883,089)	Uninsured (N = 24,300,000)	T-test	F-statistics	p-value
Age (years)	-	48.68 (18.14)	39.33 (11.07 )	-56.01	-	<0.001***
Sex	-	-	-	-	120.86	<0.001***
Male	98,474,906 (50%)	84,928,267 (49%)	13,546,639 (56%)	-	-	-
Female	99,708,183 (50%)	88,952,114 (51%)	10,756,069 (44%)	-	-	-
Educational level	-	-	-	-	1785.40	<0.001***
HS graduate	59,927,070 (30%)	57,029,494 (33%)	2,897,575 (12%)	-	-	-
Did not graduate	138,256,020 (70%)	116,850,887 (67%)	21,405,133 (88%)	-	-	-
Income	-	-	-	-	1490.49	<0.001***
Less than $15,000	50,918,274 (26%)	39,385,084 (23%)	11,533,190 (47%)	-	-	-
$15,000-$24,999	44,460,084 (22%)	37,563,283 (22%)	6,896,801 (28%)	-	-	-
$25,000-$34,999	102,804,731 (52%)	96,932,013 (56%)	5,872,718 (24%)	-	-	-
Race/ethnicity	-	-	-	-	548.95	<0.001***
Non-Hispanic White	1.2e+08 (63%)	1.1e+08 (66%)	9,701,069 (40%)	-	-	-
Non-Hispanic Black	22,933,463 (12%)	19,826,897 (11%)	3,106,567 (13%)	-	-	-
Non-Hispanic American Indian	2,092,284 (1%)	1,831,431 (1%)	260,854 (1%)	-	-	-
Non-Hispanic Asian	10,142,758 (5%)	9,062,849 (5%)	1,079,910 (4%)	-	-	-
Non-Hispanic Native Hawaiian	421,482 (<0%)	371,206 (<0%)	50,276 (<0%)	-	-	-
Non-Hispanic other race	1,093,502 (1%)	931,457 (1%)	162,045 (1%)	-	-	-
Non-Hispanic multiracial	2,615,066 (1%)	2,344,995 (1%)	270,072 (1%)	-	-	-
Hispanic	34,505,853 (17%)	24,833,937 (14%)	9671916(40%)	-	-	-

Out of an estimated population of 198,183,089, approximately 173,883,089 individuals were insured, while 24,300,000 were uninsured. Statistically significant differences were observed between the two groups across all examined characteristics (p < 0.001 for all comparisons).

The mean age of insured individuals was significantly higher than that of the uninsured group (48.7 years (SD = 18.1) vs. 39.3 years (SD = 11.1), t = -56.01, p < 0.001). In terms of sex, a larger proportion of uninsured individuals were male, 13,546,639 (56%), compared to 84,928,267 (49%) among the insured. Conversely, females represented a smaller share of the uninsured population, 10,756,069 (44%), compared to 88,952,114 (51%) among the insured (F = 120.86, p < 0.001).

Educational attainment also differed notably by insurance status. Among the uninsured, 21,405,133 individuals (88%) had not graduated from high school, in contrast to 116,850,887 (67%) in the insured group. Only 2,897,575 (12%) of the uninsured were high school graduates, compared to 57,029,494 (33%) of the insured population (F = 1785.40, p < 0.001).

Income disparities were evident. Nearly half of the uninsured, 11,533,190 (47%), reported annual incomes of less than $15,000, compared to 39,385,084 (23%) of those with insurance. Similarly, 6,896,801 (28%) of the uninsured earned between $15,000 and $24,999, versus 37,563,283 (22%) among the insured. A smaller proportion of the uninsured (5,872,718; 24%) had incomes ranging from $25,000 to $34,999, compared to 96,932,013 (56%) of the insured population (F = 1490.49, p < 0.001).

Racial and ethnic composition also varied significantly. Non-Hispanic White adults represented 114,000,000 (66%) of the insured group, but only 9,701,069 (40%) of the uninsured. In contrast, Hispanic adults comprised 9,671,916 (40%) of the uninsured population compared to 24,833,937 (14%) of the insured. Among Black, non-Hispanic individuals, 3,106,567 (13%) were uninsured versus 19,826,897 (11%) who were insured. Other racial/ethnic groups, including American Indian, Asian, Native Hawaiian, and multiracial individuals, each constituted about 1%-5% of the sample, with small but statistically significant differences across insurance status groups (F = 548.95, p < 0.001).

Table 2 shows the distribution of sociodemographic characteristics by recent checkup status among US adults.

**Table 2:** Distribution of sociodemographic characteristics by recent checkup status among US adults, BRFSS 2023 (weighted data, n = 329,549; population size = 198,183,089) Values are survey-weighted using the BRFSS 2019 complex sampling design. Categorical variables were compared using design-based F-tests; continuous variables (e.g., age) were compared using survey-weighted t-tests. All p-values are two-sided. Asterisks indicate statistical significance: p < 0.05 (*), p < 0.01 (**), p < 0.001 (***). Comparisons reflect differences between individuals with a recent checkup versus those without. HS: high school.

Characteristic	Total (weighted %)	Recent checkup (N = 151,591,198)	No recent checkup (N = 47,023,892)	T-test	F-statistics	p-value
Age (years)	-	49.77 (18.21)	40.29 (13.38)	-73.15	-	<0.001***
Insured	-	-	-	-	3923.25	<0.001***
Insured	173,880,381 (88%)	139,819,494 (92%)	34,060,887 (73%)	-	-	-
Uninsured	24,302,708 (12%)	11,511,870 (8%)	12,790,838 (27% )	-	-	-
Sex	-	-	-	-	673.44	<0.001***
Male	98,474,906 (50%)	71,128,951 (47%)	27,345,955 (58%)	-	-	-
Female	99,708,183 (50%)	80,202,413 (53%)	19,505,770 (42%)	-	-	-
Educational level	-	-	-	-	151.81	<0.001***
HS graduate	59,927,070 (30%)	47,329,389 (31%)	12,597,681 (27%)	-	-	-
Did not graduate	138,256,020 (70%)	104,001,975 (69%)	34,254,044 (73%)	-	-	-
Income	-	-	-	-	1490.49	<0.001***
Less than $15,000	50,918,274 (26%)	38,246,088 (25%)	12,672,186 (27%)	-	-	-
$15,000-$24,999	44,460,084 (22%)	33,106,685 (22%)	11,353,399 (24%)	-	-	-
$25,000-$34,999	102,804,731 (52%)	79,978,591 (53%)	22,826,140 (49%)	-	-	-
Race/Ethnicity	-	-	-	-	82.68	<0.001***
Non-Hispanic White	124,378,679 (63%)	95,949,294 (63%)	28,429,385 (61%)	-	-	-
Non-Hispanic Black	22,933,463 (12%)	19,093,605 (13%)	3,839,859 (8%)	-	-	-
Non-Hispanic American Indian	2,092,284 (10%)	1,578,683 (10%)	513,601 (10%)	-	-	-
Non-Hispanic Asian	10,142,758 (5%)	7,514,218 (5%)	2,628,541 (6%)	-	-	-
Non-Hispanic Native Hawaiian	421,482(<0%)	338,043 (<0%)	83,439 (<0%)	-	-	-
Non-Hispanic other race	1,093,502 (1%)	819,502 (10%)	274,000 (1%)	-	-	-
Non-Hispanic multiracial	2,615,066 (1%)	1,943,521 (10%)	671,546 (1%)	-	-	-
Hispanic	34,505,853 (17%)	24,094,499 (16%)	10,411,355 (22%)	-	-	-

Of the total population, an estimated 151,591,198 individuals (approximately 77%) reported having had a recent checkup, while 47,023,892 (23%) had not. There were statistically significant differences between these groups across all characteristics (p < 0.001 for all comparisons).

**Figure 1:**
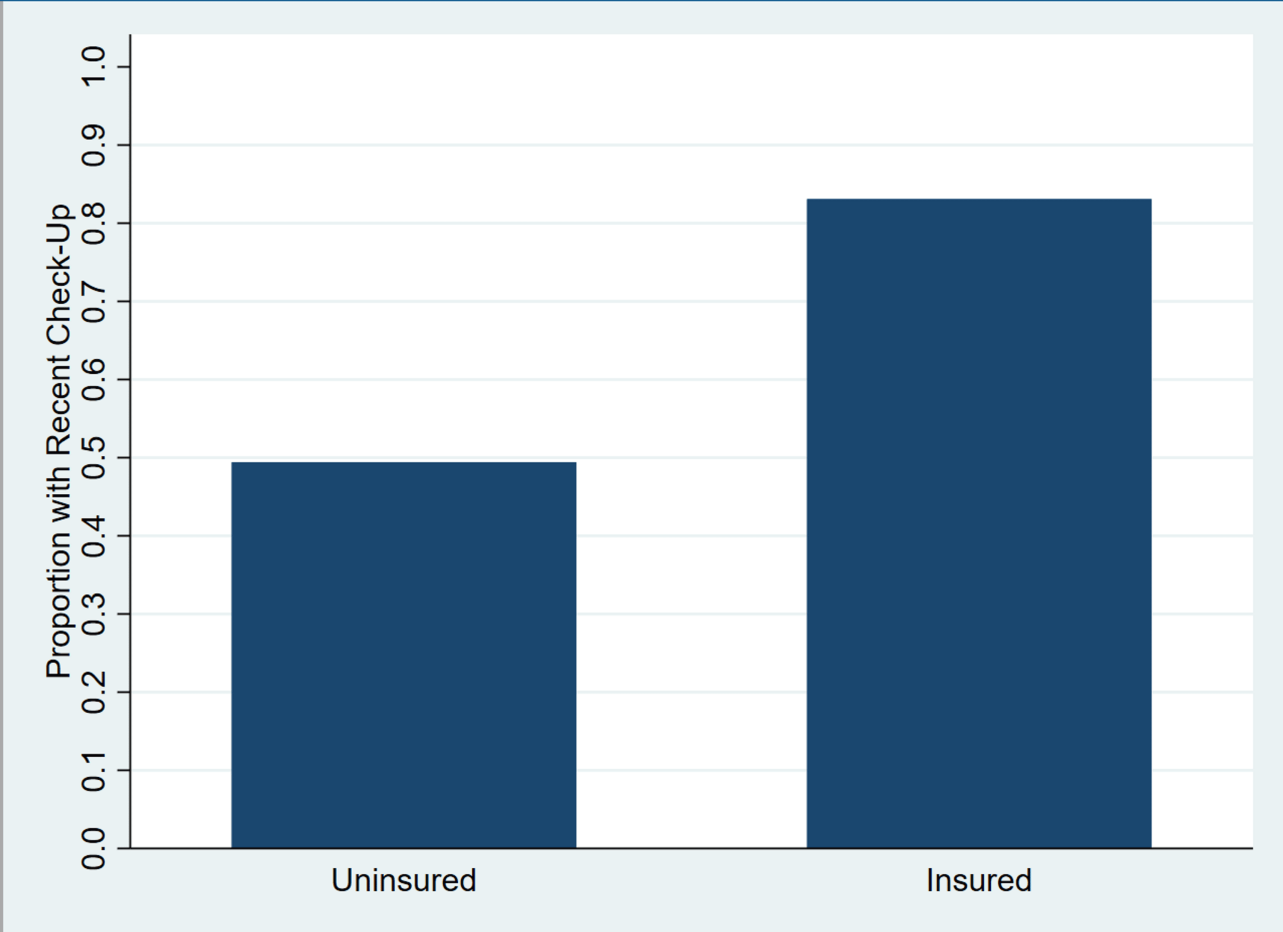
Percentage of US adults reporting a recent routine medical checkup, stratified by insurance status, BRFSS 2019 This bar chart displays the weighted proportion of US adults who reported having a routine medical checkup in the past 12 months, stratified by insurance status. Estimates are based on BRFSS 2019 data and account for the complex survey design using appropriate weights. Insured individuals were significantly more likely to have had a recent checkup compared to uninsured individuals.

Respondents who had a recent checkup were significantly older than those who did not, with a mean age of 49.8 years (SD = 18.2) compared to 40.3 years (SD = 13.4), respectively (t = -73.15, p < 0.001). Insurance status showed a marked association with recent checkup behavior. Among those with a recent checkup, 139,819,494 (92%) were insured, whereas only 11,511,870 (8%) were uninsured (Figure 1). In contrast, among individuals without a recent checkup, 12,790,838 (27%) were uninsured and 34,060,887 (73%) were insured (F = 3923.25, p < 0.001).

Gender differences were also notable. Of those who had a recent checkup, 80,202,413 (53%) were female and 71,128,951 (47%) were male. Among individuals without a recent checkup, the majority were male (27,345,955; 58%), while females accounted for 19,505,770 (42%) (F = 673.44, p < 0.001).

Educational attainment was significantly related to recent checkup utilization. Among individuals who had a recent checkup, 47,329,389 (31%) were high school graduates, and 104,001,975 (69%) had not completed high school. Similarly, of those who did not have a recent checkup, 12,597,681 (27%) were high school graduates, while a larger proportion (34,254,044; 73%) had not completed high school (F = 151.81, p < 0.001).

Income was also associated with checkup behavior. Of those with recent checkups, 38,246,088 (25%) earned less than $15,000 annually, and 33,106,685 (22%) earned between $15,000 and $24,999. Another 79,978,591 (53%) reported earning between $25,000 and $34,999. Among those without a recent checkup, 12,672,186 (27%) earned less than $15,000, 11,353,399 (24%) earned $15,000-$24,999, and 22,826,140 (49%) reported annual incomes between $25,000 and $34,999 (F = 1490.49, p < 0.001).

Racial and ethnic differences were evident as well. Among individuals with recent checkups, 95,949,294 (63%) were non-Hispanic White, 19,093,605 (13%) were non-Hispanic Black, and 24,094,499 (16%) were Hispanic. In the group without recent checkups, non-Hispanic White individuals comprised 28,429,385 (61%), non-Hispanic Black individuals 3,839,859 (8%), and Hispanics 10,411,355 (22%). Other groups, including Asian, American Indian, Native Hawaiian, and multiracial individuals, each represented 1%-6% of their respective groups, with statistically significant differences across categories (F = 82.68, p < 0.001).

Table 3 shows the AORs for predictors of having a recent medical checkup among US adults.

**Table 3:** Adjusted odds ratios for predictors of having a recent medical checkup among US adults, BRFSS 2023 (weighted logistic regression model, n = 329,549; population size = 198,183,089) Estimates are derived from a survey-weighted logistic regression model using BRFSS 2019 data. Values represent adjusted odds ratios (AORs) with 95% confidence intervals (CIs). The model controls for insurance status, age, sex, education, income, and race/ethnicity. All p-values are two-sided. Asterisks indicate statistical significance: p < 0.05 (*), p < 0.01 (**), p < 0.001 (***). Reference groups include: uninsured (insurance), male (sex), less than high school education (education), annual income < $15,000 (income), and non-Hispanic White (race/ethnicity). HS: high school

Recent checkup	Odds ratio (OR)	Std. error	95% CI (lower-upper)	p-value
Insured	3.90	0.11	3.69-4.12	<0.001***
Age	1.03	0.001	1.02-1.03	<0.001***
Female	1.52	0.03	1.47-1.58	<0.001***
Educational level	-	-	-	-
HS graduate	1.00	0.02	0.96-1.04	1.04
Income	-	-	-	-
$15,000-$24,999	0.90	0.02	0.85-0.95	0.95
$25,000-$34,999	0.98	0.03	0.92-1.03	1.03
Race/ethnicity	-	-	-	-
Non-Hispanic Black	1.86	0.07	1.73-2.00	<0.001***
Non-Hispanic American Indian	1.03	0.07	0.90-1.18	0.62
Non-Hispanic Asian	1.13	0.07	1.00-1.27	0.04*
Non-Hispanic Native Hawaiian	1.61	0.21	1.25-2.07	<0.001***
Non-Hispanic other race	1.07	0.12	0.86-1.33	0.56
Non-Hispanic multiracial	1.08	0.06	0.96-1.21	0.19
Hispanic	1.16	0.03	1.10-1.23	<0.001***

After adjusting for covariates, several factors were significantly associated with the likelihood of having had a recent medical checkup. Health insurance coverage emerged as the strongest predictor: insured individuals had nearly four times the odds of reporting a recent check-up compared to their uninsured counterparts (OR = 3.90, SE = 0.11, 95% CI: 3.69-4.12, p < 0.001). Age was also positively associated with recent checkup behavior, with each additional year of age increasing the odds by 3% (OR = 1.03, SE = 0.001, 95% CI: 1.02-1.03, p < 0.001). Female participants were significantly more likely than males to report a recent checkup (OR = 1.52, SE = 0.03, 95% CI: 1.47-1.58, p < 0.001).

In terms of socioeconomic factors, income showed mixed associations. Compared to individuals earning less than $15,000 per year, those in the $15,000-$24,999 income group had slightly lower odds of recent checkups (OR = 0.90, SE = 0.02, 95% CI: 0.85-0.95), while those earning $25,000-$34,999 had odds not significantly different from the reference group (OR = 0.98, SE = 0.03, 95% CI: 0.92-1.03). Educational level showed no significant association in this model, with high school graduates having nearly identical odds as those who did not complete high school (OR = 1.00, SE = 0.02, 95% CI: 0.96-1.04, p = 1.04).

Race and ethnicity were also significantly associated with checkup behavior. Compared to non-Hispanic White adults, non-Hispanic Black adults had significantly higher odds of having a recent checkup (OR = 1.86, SE = 0.07, 95% CI: 1.73-2.00, p < 0.001), as did Hispanic individuals (OR = 1.16, SE = 0.03, 95% CI: 1.10-1.23, p < 0.001). Asian adults had slightly higher odds compared to White adults (OR = 1.13, SE = 0.07, 95% CI: 1.00-1.27, p = 0.04), and Native Hawaiian individuals also had increased odds (OR = 1.61, SE = 0.21, 95% CI: 1.25-2.07, p < 0.001). However, American Indian adults and other non-Hispanic racial groups did not show statistically significant differences in the likelihood of recent checkups relative to White individuals. These findings emphasize the significant influence of health insurance status, age, sex, and race/ethnicity on preventive healthcare utilization, even after adjusting for socioeconomic indicators.

**Figure 2:**
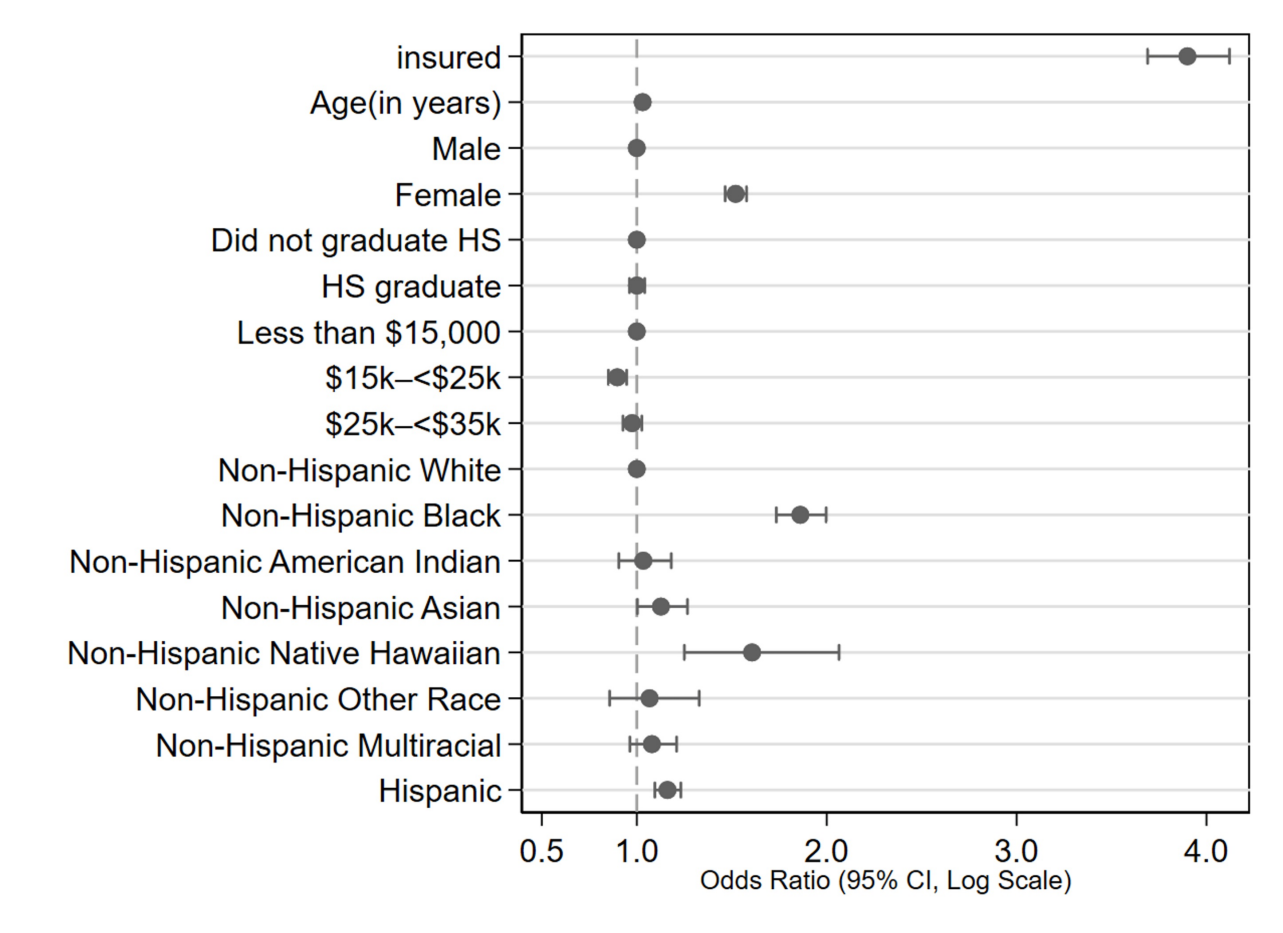
Adjusted odds ratios for predictors of recent routine medical checkup among US adults, BRFSS 2019 Forest plot displaying adjusted odds ratios (AORs) with 95% confidence intervals for factors associated with having had a routine medical checkup in the past 12 months. Estimates are derived from a survey-weighted logistic regression model controlling for insurance status, age, sex, educational level, income, and race/ethnicity. Health insurance coverage, female sex, older age, and Hispanic or Black race/ethnicity were significantly associated with higher odds of recent checkup utilization. Reference groups include: uninsured, male, did not graduate from high school, income < $15,000, and non-Hispanic White.

These findings are visually summarized in Figure 2, which presents a forest plot of AORs and 95% CIs for the likelihood of recent checkups. Insurance coverage was the strongest predictor, followed by sex and racial/ethnic background. Several income and education categories did not show statistically significant differences relative to their respective reference groups.

## Discussion

This study investigated the relationship between health insurance status and the likelihood of having a recent medical checkup among US adults using weighted 2019 BRFSS data. The findings demonstrate that health insurance coverage is critical in facilitating access to routine preventive healthcare services. Individuals with health insurance were significantly more likely to report a recent medical checkup compared to those without coverage, even after adjusting for demographic and socioeconomic characteristics. Specifically, insured adults had nearly four times the odds of a recent checkup compared to the uninsured, highlighting insurance coverage as a central enabler of preventive care utilization. Next, we summarize the key contributions of this study: quantification of the strong association between insurance status and checkup utilization, demonstration of demographic and socioeconomic gradients, and identification of persistent racial/ethnic disparities after adjustment for other covariates.

The observed association between older age and increased likelihood of a recent checkup aligns with previous research suggesting that older adults are more engaged with routine care, possibly due to higher health needs or Medicare eligibility. Additionally, female participants were significantly more likely than males to report recent checkups. This sex difference is consistent with prior studies, indicating that women are more proactive in seeking preventive care, possibly due to reproductive health service needs and greater health-seeking behavior overall.

Socioeconomic factors also influenced healthcare utilization. Similar socioeconomic gradients have been documented internationally, for example, in Thailand, where high‑income, highly educated, insured, and urban residents were significantly more likely to use annual health checkups, and in the United States, where lower‑income and less‑educated women exhibited lower rates of mammography screening both before and after the COVID‑19 pandemic [22,23]. While education did not show a statistically significant association in the multivariate model, income did: individuals in the lower‑middle income range ($15,000-$24,999) were less likely to have had a recent checkup compared to those in the lowest income group. This unexpected finding may be reflective of nuanced barriers within working‑class populations, such as lack of paid time off, underinsurance, or gaps in Medicaid expansion coverage. Furthermore, racial and ethnic disparities were evident. Non‑Hispanic Black and Hispanic adults were more likely to report recent check‑ups than non‑Hispanic Whites, even after adjusting for insurance and socioeconomic factors. These findings suggest that while disparities in healthcare access persist, targeted outreach or culturally specific healthcare delivery models may be effectively reaching some underserved communities.

The study’s findings align with the broader literature documenting the positive impact of health insurance on preventive service use. Systematic reviews have shown that community‑based and other health insurance schemes significantly increase the utilization of preventive and primary care services by two‑ to threefold, and insurance coverage has been linked to higher rates of antenatal visits, facility delivery, and skilled birth attendance across low‑ and middle‑income settings [24,25]. Our results add to this evidence base using recent, nationally representative data, reinforcing the critical importance of expanding and maintaining health insurance coverage as a public health strategy.

The insights provided by this study may be used by the primary care physicians (PCPs) to create targeted outreach strategies addressing the barriers faced by uninsured and low-income populations in accessing preventive care. Owing to the observation that individuals without health insurance are less likely to go for regular check-ups, PCPs might prioritize community-based education initiatives that aim to educate such populations on the accessibility and value of preventive care.

Logistical and fiscal challenges might additionally be solved through putting in place sliding-scale charge structures, provision of treatments at the community health fairs, and through collaboration with local organizations to deliver culturally competent care [26]. PCPs may also utilize socioeconomic determinants of health screening tools to locate and find patients who might be underutilizing care. They might then arrange follow-up with patient navigators and mobile health clinics [27]. These initiatives improve access and foster trust and care continuity within underserved populations, eventually enhancing early detection and management of chronic diseases.

Limitations

However, the study has several limitations. The most significant limitation is the handling of missing data. We adopted a complete case analysis approach, excluding all records with missing values on key analytic variables. Although this method simplifies analysis and maintains consistency across models, it can introduce bias if the missingness is not completely at random. Notably, approximately 18% of cases had missing income data, potentially affecting the accuracy and generalizability of income-related findings. Future studies should consider multiple imputation or other robust methods to address missing data, especially when using complex survey datasets like BRFSS.

Another limitation is the cross-sectional nature of the BRFSS data, which precludes any causal inferences. The associations observed do not establish temporal direction or causality. Additionally, all data were self-reported, which introduces potential for recall bias or social desirability bias, particularly regarding insurance status and preventive health behavior.

Despite these limitations, this study provides timely and policy-relevant insights into factors influencing preventive care use in the US adult population. The results emphasize the importance of health insurance as a gateway to routine medical care and highlight demographic disparities that warrant continued public health attention. As healthcare policy evolves, ensuring broad, equitable access to health coverage remains essential to improving population health outcomes. Future research should consider retrospective cohort or longitudinal designs to establish temporal relationships between insurance changes and preventive care utilization.

## Conclusions

This study demonstrates that health insurance coverage is a key determinant of routine medical checkup utilization among US adults. Insured individuals were nearly four times more likely to report having had a recent checkup compared to their uninsured counterparts. This association remained significant after adjusting for age, sex, income, education, and race/ethnicity, highlighting the essential role of insurance in facilitating access to preventive healthcare.

Sociodemographic disparities were also observed. Female respondents, older adults, and individuals identifying as Hispanic or non-Hispanic Black were more likely to have had recent checkups, while those with lower income and educational attainment were less likely to do so. However, the relationship between checkup rates and socioeconomic status was not entirely linear, suggesting that additional structural or behavioral barriers may influence care-seeking behavior. These findings emphasize the need for public health policies that expand and sustain comprehensive insurance coverage while addressing underlying disparities. Targeted outreach to vulnerable groups and further research into access barriers are necessary to improve preventive care utilization and promote health equity nationwide.
